# Case Report: Isolated leptomeningeal relapse after systemic control in PALB2-altered pancreatic cancer

**DOI:** 10.3389/fmed.2026.1927688

**Published:** 2026-10-12

**Authors:** Jian Yuan, Yangxian Xu, Qihao Zhou, Guorong Yuan, Meizhen Ye

**Affiliations:** 1 Department of General Surgery, Longhua Hospital, Shanghai University of Traditional Chinese Medicine, Shanghai, China; 2 Cancer Center, Department of Medical Oncology, Zhejiang Provincial People’s Hospital (Affiliated People’s Hospital, Hangzhou Medical College), Hangzhou, Zhejiang, China

**Keywords:** case report, cerebrospinal fluid sequencing, CNS sanctuary relapse, homologous recombination repair, leptomeningeal metastasis, PALB2, pancreatic ductal adenocarcinoma

## Abstract

**Background:**

Leptomeningeal metastasis (LM) is an uncommon manifestation of pancreatic ductal adenocarcinoma (PDAC) and has a median survival of only 1–2 months after diagnosis. Isolated LM after prolonged extracranial disease control is particularly rare and may be overlooked when systemic imaging results look stable.

**Case presentation:**

We describe a 51-year-old man with metastatic PDAC who initially received a treatment regimen comprising KN046 and gemcitabine and nab-paclitaxel, followed by a prolonged course of folinic acid, fluorouracil, irinotecan, and oxaliplatin (FOLFIRINOX), and subsequently underwent conversion surgery with a pathological complete response (ypT0N0, R0). Several months after completing adjuvant FOLFIRINOX, the patient developed a new right lower limb weakness. Brain MRI and cerebrospinal fluid (CSF) cytology results confirmed the presence of isolated LM, but there was no evidence of active extracranial disease. CSF sequencing identified Kirsten rat sarcoma viral oncogene homolog G12D, an AT-rich interaction domain 1A frameshift alteration, and a truncating partner and localizer of BRCA2 p.L920* alteration involving the homologous recombination repair (HRR) pathway. Then, the patient underwent ventriculoperitoneal shunt and Ommaya reservoir placement, followed by treatment with serial intrathecal methotrexate plus dexamethasone and systemic toripalimab with bevacizumab. This multimodal approach was followed by temporary neurological stabilization, but the disease eventually progressed. He survived approximately 30 months since the initial PDAC diagnosis and 3.5 months after LM diagnosis.

**Conclusion:**

Together with our review of 29 published PDAC-LM cases, this report suggests two recurring clinical patterns: fulminant presentation at/or near initial diagnosis and delayed central nervous system (CNS) relapse after prolonged systemic disease control. The repeated detection of Fanconi anemia/homologous recombination repair pathway alterations in several genomically characterized cases gives rise to the possibility that treatment-responsive, HRR pathway–altered PDAC may survive long enough for CNS sanctuary relapse to become clinically apparent. CSF genomic profiling may help characterize rare CNS relapse in molecularly defined PDAC, and new neurological symptoms in long-surviving patients warrant prompt evaluation even when extracranial disease appears controlled.

## Introduction

Pancreatic ductal adenocarcinoma (PDAC) has a 5-year survival rate of only approximately 13% and remains one of the most lethal solid tumors (1). Metastases most often involve the liver, peritoneum, and lungs, whereas involvement of the central nervous system (CNS) is uncommon (2). Brain metastases occur in only 0.25%–0.6% of patients with PDAC (2, 3), although autopsy data suggest an incidence rate as high as 7.9%, indicating significant underdiagnosis (4). As systemic therapy has extended survival rates in advanced PDAC (5), longer disease courses may widen the window for CNS relapse (4, 6).

Leptomeningeal metastasis (LM) refers to the dissemination of malignant cells within the cerebrospinal fluid (CSF) and leptomeninges. This frequently fatal complication of advanced solid tumors has an estimated median survival of 2–6 months despite multimodality treatment (3, 7, 8). Among patients with PDAC, LM is extremely rare and has a particularly dismal prognosis, with a reported median survival of only 1.6 months after diagnosis (9–11). Most reported cases of PDAC-related LM have occurred in the setting of prolonged systemic disease control, with LM emerging as an isolated pattern of CNS relapse, consistent with the sanctuary site hypothesis (6, 12). Optimal management of PDAC-related LM remains uncertain, particularly in patients without evidence of active extracranial disease (3). Current European Association of Neuro-Oncology–European Society for Medical Oncology (EANO-ESMO), and Society for Neuro-Oncology–American Society of Clinical Oncology (SNO-ASCO) guidelines recommend individualized treatment according to disease burden and the molecular profile, while acknowledging the limited evidence in this regard (3, 9). Options range from best supportive care to combinations of CSF diversion, intrathecal chemotherapy, and systemic therapy (13).

Herein, we describe a patient with metastatic PDAC who achieved a pathological complete response (pCR) after systemic chemotherapy and conversion surgery. The patient subsequently developed isolated LM, although extracranial disease remained controlled. This patient was managed with ventriculoperitoneal (VP) shunting, intrathecal methotrexate (MTX) that was administered via an Ommaya reservoir, and systemic immunotherapy combined with antiangiogenic therapy. His overall survival was approximately 30 months since initial PDAC diagnosis and 3.5 months after LM diagnosis, exceeding the median outcomes reported for PDAC-related LM. We present his clinical course, interpret the CSF molecular findings, and compare his disease trajectory with previously reported PDAC-LM cases.

## Case description

The patient was a 51-year-old man with no significant past medical history and no notable alcohol or tobacco use. There was no reported family history of cancer or hereditary disease. This patient presented to our hospital with upper abdominal pain (month 1; all later time points are referenced to the month of initial PDAC diagnosis). A contrast-enhanced abdominal CT scan showed a pancreatic mass with multiple liver metastases (Figures 1A,B), and a liver biopsy confirmed the presence of metastatic pancreatic ductal adenocarcinoma. Within a phase III clinical trial, the patient received seven cycles of first-line therapy with a regimen comprising an anti-PD-L1/CTLA-4 bispecific antibody (KN046), nab-paclitaxel, and gemcitabine over 6 months (months 1–7), achieving a partial response. The hepatic metastases then progressed after a period of control (Figures 1C,D). At month 8, treatment was switched to modified folinic acid, fluorouracil, irinotecan, and oxaliplatin (mFOLFIRINOX) and continued for 17 cycles through month 16, again meeting with a partial response.

**Figure 1:**
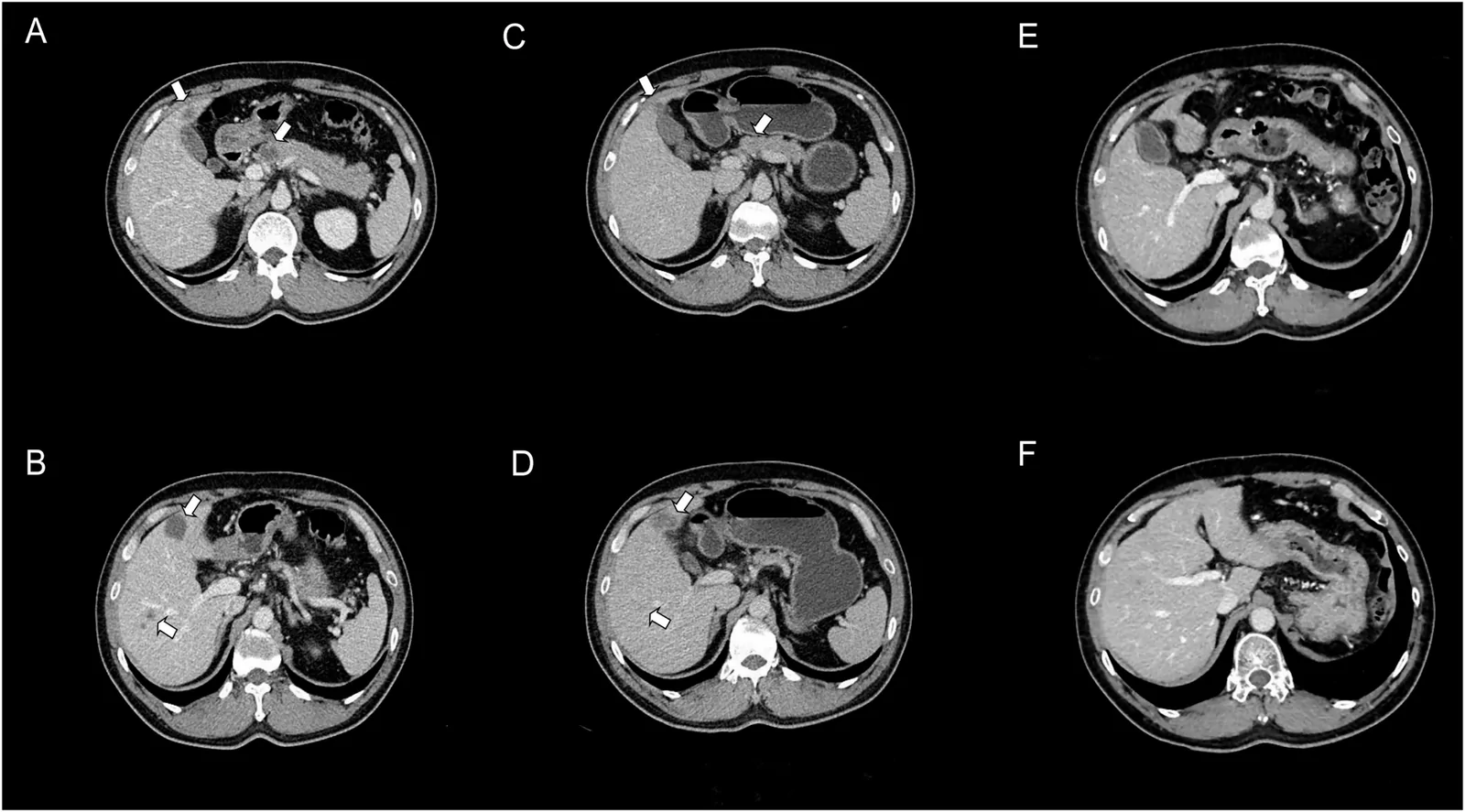
Contrast-enhanced abdominal CT images showing the radiographic course of the pancreatic primary tumor and hepatic metastases. **(A,B)** At initial presentation (month 1), a CT scan demonstrated the pancreatic primary lesion together with multiple hepatic metastases. **(C,D)** After first-line therapy (month 7), the hepatic metastases showed partial progression. **(E,F)** After surgery and subsequent systemic therapy (month 26), no radiographically evident intra-abdominal disease was identified. The arrows indicate representative lesions.

In month 17, the patient underwent laparoscopic radical distal pancreatectomy along with splenectomy. Pathology results showed a complete response at the primary site (ypT0N0, R0; CAP tumor regression grade 0). Then, he received 16 cycles of postoperative mFOLFIRINOX over the next 8 months, and restaging imaging results showed no radiographically evident intra-abdominal disease (Figures 1E,F). At month 27, a CT scan showed an indeterminate lesion in hepatic segment IV adjacent to the gallbladder fossa. A positron emission tomography–computed tomography scan performed at the same time showed no increased fluorodeoxyglucose uptake or definite evidence of recurrence or metastasis. After a multidisciplinary team discussion, laparoscopic partial hepatectomy was performed, and histopathology results showed treatment-related fibrosis without residual malignancy.

### Diagnostic assessment

One month later (month 28), the patient developed new right lower limb weakness [Medical Research Council (MRC) grade III] without other focal neurological deficits. Brain MRI results showed abnormal leptomeningeal enhancement, predominantly in the left frontoparietal region, with adjacent parenchymal signal abnormality (Figures 2A,B). Lumbar puncture supported leptomeningeal involvement: an opening pressure of 190 mmH_2_O, a markedly elevated CSF protein level (115.4 mg/dL), elevated levels of lactate dehydrogenase (LDH; 45 U/L) and lactate (7.0 mmol/L), reduced glucose level (1.72 mmol/L), and a carcinoembryonic antigen (CEA) level of 347.6 µg/L. A CSF cytology analysis identified malignant cells (5% of nucleated cells), confirming leptomeningeal metastasis from PDAC. Targeted next-generation sequencing (NGS) of the CSF identified a truncating partner and localizer of BRCA2 (PALB2) p.L920* mutation, an AT-rich interaction domain 1A (ARID1A) frameshift mutation, and a Kirsten rat sarcoma viral oncogene homolog (KRAS) p.G12D driver mutation.

**Figure 2:**
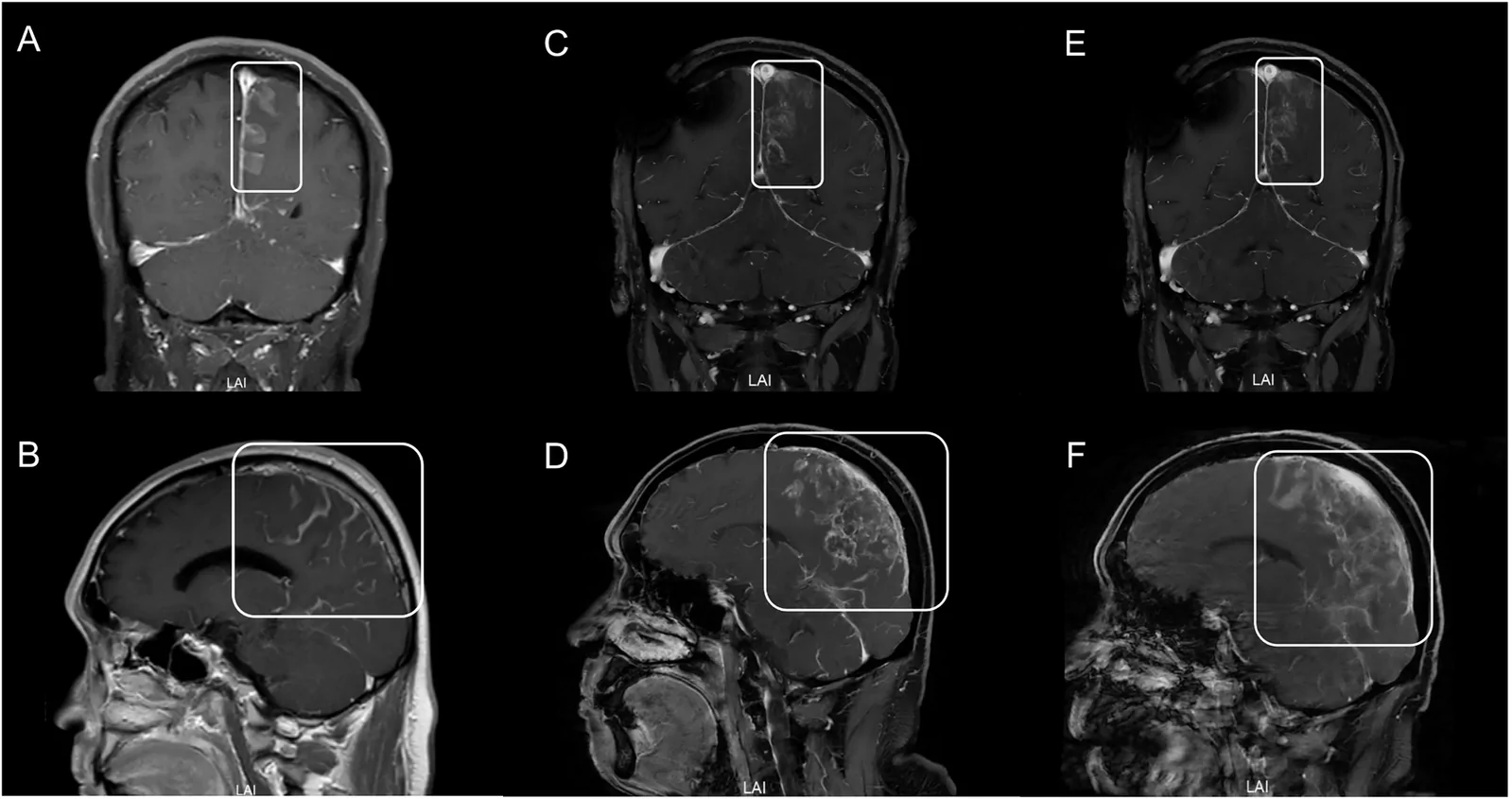
A contrast-enhanced brain MRI showing the radiographic evolution of leptomeningeal metastasis. **(A,B)** At the initial diagnosis of leptomeningeal metastasis (month 28), MRI showed abnormal leptomeningeal enhancement in the left frontoparietal region. **(C,D)** On follow-up MRI after intrathecal therapy (month 29), diffuse leptomeningeal enhancement remained extensive, with slight interval progression compared with that observed in the baseline study. **(E,F)** At terminal progression (month 31), MRI demonstrated a marked progression of leptomeningeal disease. The boxed areas indicate representative regions of abnormal enhancement.

### Therapeutic intervention

At month 28, the patient underwent a right VP shunt placement with laparoscopic assistance and placement of a left lateral ventricular Ommaya reservoir. These procedures provided CSF diversion and access to intraventricular therapy. During months 28–30, the patient received 14 intrathecal administrations of methotrexate (10 mg) and dexamethasone (5 mg) via the Ommaya reservoir. Treatment was administered at intervals of 3–8 days over approximately 9 weeks without dose adjustment and was well tolerated. Following a multidisciplinary team discussion, the patient concurrently received the anti-PD-1 antibody toripalimab (240 mg) and bevacizumab (7.5 mg/kg) intravenously every 3 weeks for four cycles. Toripalimab was selected because the previous KN046-containing regimen had produced a partial response and was well tolerated. Bevacizumab was included for edema control and potential synergy with PD-1 blockade.

### Follow-up and outcomes

Weakness in the right lower limb of the patient initially worsened after surgery (MRC grade I), accompanied by drowsiness and incoherent speech, but the patient began to regain consciousness approximately 1 week into intrathecal therapy and regained limb strength to MRC grade III. Serial CSF analyses showed a marked fall in nucleated cell counts and inflammatory mediator levels, including interleukin-6 (IL-6) and interleukin-8 (IL-8); protein, CEA, and LDH levels fluctuated but generally improved during the early treatment period (Supplementary Figures S1A–F). At the 29-month follow-up, the patient remained alert and there was further reduction in neurological symptoms. However, brain MRI results continued to show diffuse leptomeningeal enhancement (Figures 2C,D). The overall course of treatment is summarized in Figure 3.

**Figure 3:**
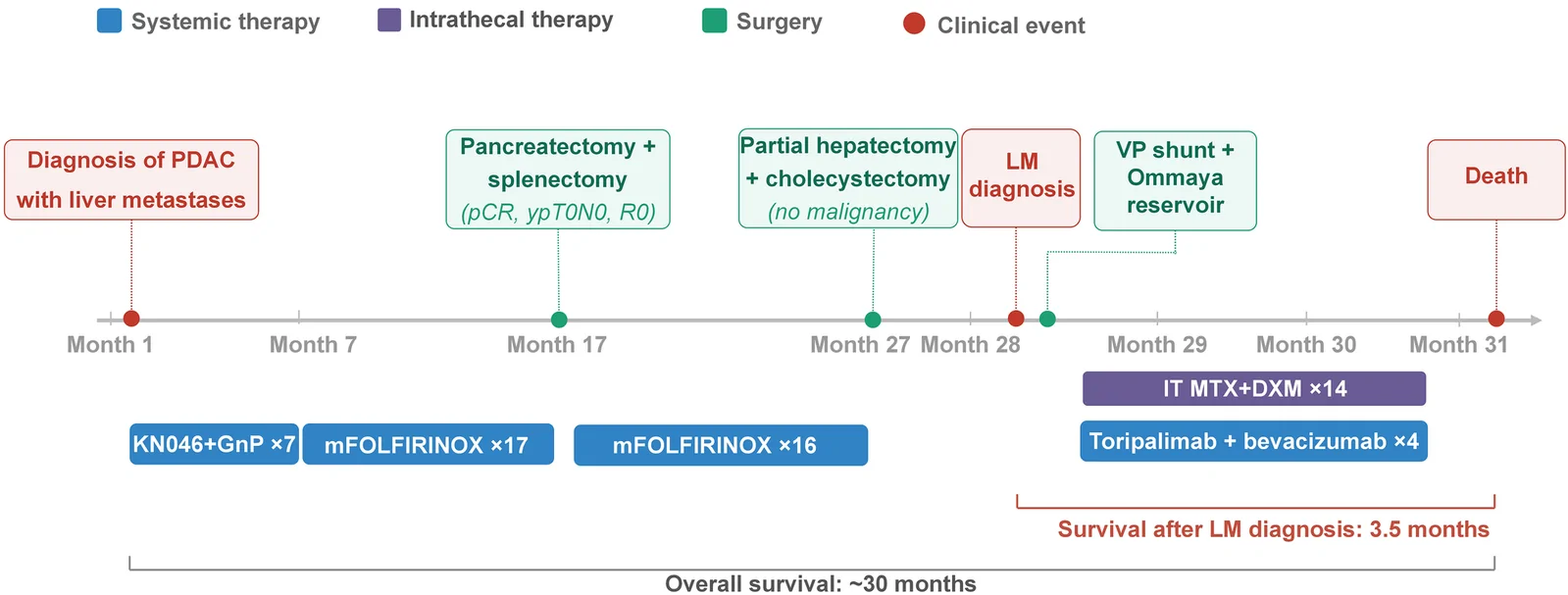
A clinical timeline of systemic, surgical, and CNS-directed treatments. PDAC, pancreatic ductal adenocarcinoma; LM, leptomeningeal metastasis; pCR, pathological complete response; VP shunt, ventriculoperitoneal shunt; MTX, methotrexate; DXM, dexamethasone.

By month 31, the patient had recurrent drowsiness, dysphagia, and fever. Brain MRI results showed enlarging leptomeningeal lesions with worsening cerebral edema, consistent with progression (Figures 2E,F). The patient died later that month of respiratory failure secondary to severe pneumonia, approximately 30 months after the initial PDAC diagnosis and 3.5 months after the LM diagnosis.

## Discussion

We searched PubMed, Google Scholar, and journal websites for published reports of PDAC with LM. Search terms combined “pancreatic cancer” or “pancreatic adenocarcinoma” with “leptomeningeal metastasis”, “leptomeningeal carcinomatosis”, or “carcinomatous meningitis”. The last literature search was performed on 23 April 2026. We included case reports and case series describing PDAC with LM without language or publication date restrictions. Reports of brain parenchymal metastases without LM were excluded from Table 1.

**Table 1:** Summary of reported PDAC cases with leptomeningeal metastasis.

Reference	Sex/age	Extracranial disease at LM diagnosis	Systemic therapy	CNS therapy	OS (months)	Post-LM survival (months)	Genomic alterations
Al Zarkali et al. (27)	M/75	None	mFOLFIRINOX	Craniotomy + CSI	18	3	NR
Chemarthi et al. (28)	M/50	Liver	FOLFIRINOX, GnP, and irinotecan + bevacizumab	Focal RT (skull base)	NR	1	NR
Walker et al. (19)	M/50	Peritoneal	FOLFIRINOX, capecitabine, olaparib, and GEM + cisplatin	None	>57	<1	PALB2, CDKN2A, KRAS, SMAD4, and TP53
Seu et al. (29)	M/72	Locally advanced	FOLFIRINOX, capecitabine + RT	WBRT	18	6	NR
Sayanagi et al. (18)	F/49	Hilar and mediastinal LN	GEM + tegafur/gimeracil/oteracil, GnP, and mFOLFIRINOX	IT MTX (Ommaya)	24	<1	NR
Yim and Leung (21)	F/late 50s	Liver	mFOLFIRINOX, olaparib, and pembrolizumab	Palliative RT	11	<1	BRIP1
DeVito et al. (30)	F/43	Liver, LN, and pulmonary lymphangitic	FOLFIRINOX, ipilimumab + nivolumab, GnP + trastuzumab, and lapatinib + trastuzumab	SRS; IT trastuzumab	11	<1	KRAS G12D, TP53 W146, ERBB2, and MSI-H
Iwatsuka et al. (31)	F/57	Locally advanced	GnP	WBRT	5	5	NR
O'Connor et al. (case 1) (5)	F/61	Lung, LN, bone, and soft tissue	Gemcitabine + capecitabine + docetaxel, and GnP	None	149	0.5	KRAS G12D and ATM
O'Connor et al. (case 2) (5)	M/68	Lung, LN, adrenal and bone	FOLFIRINOX, CTLA-4, and CCR4 inhibitors (trial)	None	111	2.8	KRAS G12D, TP53, DNMT3A, NTRK1, PTPRT, and POLE
O'Connor et al. (case 3) (5)	F/57	Lung	FOLFIRINOX and GnP	None	18	2.1	KRAS G12R, TP53, MYC, CDKN2B, CDKN2Ap, and SMAD4
O'Connor et al. (case 4) (5)	M/65	Lung, LN, and peritoneum	GnP + PD-L1 and mFOLFIRINOX	Proton CSI	19	1.1	NR
Ceccon et al. (32)	M/51	Kidney, spleen, and lung	GEM + erlotinib, FOLFIRINOX, and GnP	NR	35	3	NR
Farahvash et al. (33)	F/69	Bone, mediastinal LN, lung, and peritoneum	GEM, FOLFIRINOX	WBRT and spinal RT	111	3	NR
Ikeda et al. (34)	M/59	Liver	GnP and mFOLFIRINOX	None	14	<1	NR
Johnson et al. (35)	M/53	Liver	FOLFIRINOX, FOLFIRI, and CAPIRI + bevacizumab	WBRT and IT topotecan (Ommaya)	40	10	NR
Trinh et al. (7)	M/58	None	NR	None	1	1	NR
Amico et al. (case 1) (20)	F/42	Liver and LN	GEM; PARP inhibitor (trial)	Steroids and WBRT	20	1	BRCA2 9631delC
Amico et al. (case 2) (20)	M/57	Liver, lung, and LN	Cisplatin + GEM	WBRT	15	4	BRCA1
Naqvi and Ahmed (16)	F/58	None	None	None	<1	<1	NR
Yoo et al. (6)	M/80	Liver	NR	WBRT	NR	NR	NR
Hong et al. (36)	F/72	Liver	Reolysin + carboplatin + paclitaxel	None	9	2	NR
Rao et al. (37)	M/57	Bone	FOLFIRINOX	WBRT and palliative RT	NR	NR	NR
Blows et al. (14)	M/72	Liver and LN	None	None	<1	<1	NR
Minchom et al. (15)	M/59	Locally advanced	Gemcitabine	IT MTX + Ara-C + hydrocortisone	<1	<1	NR
Hirota et al. (38)	M/64	Locally advanced	GEM + S-1	WBRT	42	40.5	NR
Rebischung et al. (39)	F/44	None	FolFuGemox, 5-FU/RT, and GemOX	WBRT, IT MTX, thiotepa, and MTX-125IUdR	44	8	NR
Grira et al. (17)	M/55	None	None	None	1.5	1.5	NR
Filho et al. (8)	M/49	Ribs, LN	Topotecan and GEM	IT MTX + Ara-C + thiotepa	6	1.5	NR
Present case	M/51	None	KN046 + GnP, mFOLFIRINOX, and toripalimab + bevacizumab	IT MTX + DXM (VP shunt and Ommaya)	30	3.5	PALB2 p.L920* (HRR pathway), ARID1A fs, and KRAS G12D

PDAC, pancreatic ductal adenocarcinoma; LM, leptomeningeal metastasis; OS, overall survival; NR, not reported; LN, lymph node; GnP, gemcitabine plus nab-paclitaxel; GEM, gemcitabine; 5-FU, fluorouracil; S-1, tegafur/gimeracil/oteracil; CAPIRI, capecitabine plus irinotecan; GemOX, gemcitabine plus oxaliplatin; WBRT, whole-brain radiotherapy; RT, radiotherapy; CSI, craniospinal irradiation; SRS, stereotactic radiosurgery; IT, intrathecal; MTX, methotrexate; DXM, dexamethasone; Ara-C, cytarabine; MSI-H, microsatellite instability-high; CCR4, C-C chemokine receptor 4; PARP, poly(ADP-ribose) polymerase; HRR, homologous recombination repair.

Leptomeningeal metastasis is rare in PDAC, and therefore, the literature is largely limited to isolated case reports and small series. We identified 29 previously published cases, which are summarized in Table 1. Among reports with survival data, the median overall survival since initial PDAC diagnosis was 18 months, whereas the median survival after LM diagnosis was only 1.5 months. Our patient survived 3.5 months after LM diagnosis, with serial neurological, MRI, and CSF assessments documenting transient clinical improvement followed by progression. The case is also distinctive for its molecular and clinical context: PALB2-altered metastatic PDAC with durable extracranial control and a pathological complete response before isolated CNS relapse.

Our review points to two recurring clinical patterns that are distinguished by the timing of LM, preceding extracranial disease control, and the predominant site of progression. Fulminant presentation refers to LM at/or near PDAC diagnosis, without prior systemic control and with rapid neurological deterioration. Trinh et al. (7) described LM as a presenting feature of pancreatic cancer. Blows et al. (14), Minchom et al. (15), and Naqvi and Ahmed (16) reported death within 1 month of LM diagnosis without prior systemic control. Grira et al. (17) reported a fulminant course lasting 6 weeks. Delayed CNS-dominant relapse refers to the emergence of LM as the dominant or isolated site of progression after a documented period of extracranial disease control. Both Sayanagi et al. (18) and Walker et al. (19) described the emergence of LM after prolonged systemic treatment and sustained extracranial control. In the Memorial Sloan Kettering series, LM developed 16–148 months after PDAC diagnosis in all four patients (5). The intra-abdominal disease burden was relatively low when LM became apparent. These reports illustrate delayed LM after prolonged systemic control as one clinical pattern within the published PDAC-LM literature. Even in this second group, post-LM survival was usually short, most often 1–3 months, and therefore, prognosis remains poor once leptomeningeal dissemination is established. Our patient clearly fits this second pattern: isolated LM emerged only after prolonged systemic treatment and durable extracranial control, with no radiographically evident extracranial disease at diagnosis.

Most PDAC-LM cases published since 2016 fall into this second pattern, with well-controlled extracranial disease and LM as an isolated or CNS-dominant relapse. This may reflect CNS sanctuary relapse, in which microscopic disease persists despite effective extracranial treatment because conventional systemic agents poorly penetrate the CNS (3). The distinction matters clinically. When PDAC is first diagnosed and a patient complains of unexplained neurological issues, LM belongs to the differential category because deterioration can be swift. In patients with durable extracranial control, new neurological symptoms still warrant careful evaluation even when systemic imaging results look stable. Current evidence does not support routine CNS surveillance in PDAC, but it does support a lower threshold for neurological evaluation in selected patients.

The delayed relapse pattern provides a clinical context for interpreting the molecular findings of the CSF in our patient. CSF sequencing identified a truncating PALB2 p.L920* alteration. Walker et al. reported a patient with PALB2-altered PDAC who developed LM after years of treatment (19). Amico et al. described two breast cancer susceptibility gene (BRCA)-associated cases: one received gemcitabine followed by a PARP inhibitor, and the other received cisplatin-based chemotherapy (20). A rapid clinical decline followed LM diagnosis in both patients. Yim and Leung reported a similar deterioration post-LM diagnosis in the condition of a patient with BRCA1-interacting protein 1 (BRIP1) mutation who received mFOLFIRINOX followed by olaparib-based therapy (21). These cases differ in treatment and survival, but PALB2, BRIP1, BRCA1, and BRCA2 all encode proteins in the Fanconi anemia/homologous recombination repair (HRR) pathway. Several HRR-altered PDAC-LM cases may therefore share a phenotype of prolonged systemic control under platinum-based or PARP-directed therapy, followed by rapid decline once LM is diagnosed.

In PDAC, pathogenic BRCA1/2 and PALB2 alterations have been associated with greater sensitivity to platinum-based chemotherapy and, in selected patients, to PARP inhibitors (22–25). Platinum sensitivity may permit prolonged extracranial control and sufficient survival for CNS relapse to become apparent. Limited drug penetration across the blood–brain and blood–CSF barriers may allow microscopic leptomeningeal disease to persist despite systemic control (3). Our case is consistent with this picture: after first-line therapy with KN046 and gemcitabine and nab-paclitaxel (GnP) and a prolonged course of FOLFIRINOX, conversion surgery showed a pathological complete response, yet leptomeningeal disease later became clinically apparent and then progressed rapidly. The PALB2 alteration may have contributed to the platinum sensitivity.

CNS-directed treatment may also have shaped the patient’s course. Once symptomatic intracranial hypertension developed, VP shunting and Ommaya reservoir placement allowed intrathecal methotrexate and dexamethasone administration and serial CSF monitoring. Bander et al. found that CSF diversion relieved symptoms in 83% of patients and enabled further oncologic treatment in 56% of a 190-patient series (26). In our patient, neurosurgical stabilization similarly allowed continued CNS-directed treatment and was followed by transient neurological stabilization, although the contribution of each intervention cannot be differentiated.

Changes in CSF indices did not in parallel match the radiographic course. After VP shunting and intrathecal therapy, nucleated cell counts of the CSF declined, whereas protein levels decreased with fluctuations. The IL-6 level fell from 408.9 to 14.1 pg/mL, and that of IL-8 fell from 684.9 to 64.1 pg/mL. The patient’s consciousness improved, and there was partial recovery in motor strength during the same period. Diffuse leptomeningeal enhancement persisted on a follow-up MRI scan at month 29 (Figures 2C,D), with radiographic and neurological progression by month 31. The serum CA19-9 level increased to >9,000 U/mL after an initial decline. No single CSF parameter reliably captured treatment response, and intermittently negative cytology in a low-cellularity sample should not be read as leptomeningeal clearance. EANO-ESMO and SNO-ASCO guidelines (3, 9) appropriately call for multidimensional assessment integrating the neurological status, imaging, CSF findings, and extracranial disease. The recovery of consciousness and motor function represented temporary clinical benefit without a clear radiographic response. CSF diversion and the anti-inflammatory effects of dexamethasone may have contributed to symptom relief and changes in CSF indices (3, 9). The later neurological and radiographic deterioration showed that early symptomatic improvement was not accompanied by sustained disease control.

This report has several limitations. As a single-case report, it cannot establish how much tumor biology, platinum sensitivity, VP shunting, intrathecal chemotherapy, or concurrent systemic therapy each contributed to the observed post-LM survival. The published literature on PDAC-LM is limited by publication bias, incomplete reporting, and heterogeneity in disease stage, treatment era, molecular testing, and follow-up. Therefore, the clinical patterns and recurrence of HRR pathway alterations described here are descriptive, hypothesis-generating observations rather than definitive findings. PALB2 p.L920* alteration was identified by CSF sequencing, but matched germline and tumor tissue sequencing were not performed. Its origin (whether germline or somatic) and presence in extracranial lesions remain unknown. The CSF finding alone does not establish functional HRR deficiency. Because the VP shunt lacked a programmable valve and remained continuously open, the dwell time and local exposure of intrathecal methotrexate may have been reduced.

In summary, leptomeningeal metastasis should be considered in long-surviving patients with PDAC who develop new neurological symptoms, even when extracranial disease appears radiographically controlled. Reported PDAC-LM courses include fulminant presentation at/or near PDAC diagnosis and delayed CNS-dominant relapse after prolonged systemic control. Our patient's disease course illustrates the latter of these clinical patterns. In selected cases, CSF diversion, intrathecal therapy, and close reassessment may offer temporary neurological stabilization, although prognosis remains poor once LM is established. Treatment-responsive PDAC with HRR pathway alterations may provide a setting in which CNS sanctuary relapse becomes apparent after prolonged extracranial control.

## Patient perspective

Throughout the treatment period, the patient and their family valued quality of life over further prolongation of survival, and intrathecal chemotherapy was well tolerated. According to his family, the period of neurological improvement was meaningful because it enabled them spend time together, while he remained relatively lucid. After the patient’s condition worsened, his family declined further antitumor therapy, and they did not regret the decision to pursue active CNS-directed treatment.

## Data Availability

The original contributions presented in the study are included in the article/Supplementary Material, and further inquiries can be directed to the corresponding author.
